# Evaluating the changes in molar incisor hypomineralization prevalence: A comparison of two cross‐sectional studies in two elementary schools in Mexico City between 2008 and 2017

**DOI:** 10.1002/cre2.252

**Published:** 2019-11-07

**Authors:** Maria Esther Irigoyen‐Camacho, Teresa Villanueva‐Gutierrez, Antonio Castano‐Seiquer, Nelly Molina‐Frechero, Marco Zepeda‐Zepeda, Leonor Sánchez‐Pérez

**Affiliations:** ^1^ Health Care Department Metropolitan Autonomous University‐Xochimilco Mexico City Mexico; ^2^ Faculty of Dentistry University of Seville Seville Spain

**Keywords:** community pediatric dentistry, dental caries, maternal perception, molar incisor hypomineralization

## Abstract

**Background:**

Little information is available on the trends over time of the prevalence of molar incisor hypomineralization (MIH). This condition may be preventing dental caries decline.

**Aim:**

(a) To compare the prevalence of MIH, in Mexico City schoolchildren, evaluated in 2008 with a group evaluated in 2017, (b) to identify the association of MIH with dental caries, and (c) to assess the mother's perception of her child's oral health status.

**Design:**

Two cross‐sectional studies performed in 2008 and in 2017 were compared. The oral examiner and the selected schools were the same in both surveys.

**Results:**

A total of 549 schoolchildren were evaluated. The prevalence of MIH in the first survey was 20.3%, and 31.9 % in the second survey, (*p* = .002). Children with MIH were more likely to have dental caries. The odds ratio was 2.24 (*p* = .036) and 4.18 (*p* ˂ .001) in the first and second surveys, respectively. Mothers of children with MIH perceived worse oral health status of their children than the mothers whose children did not have MIH (odds ratio = 4.47, *p* ˂ .001).

**Conclusion:**

The findings portray a clear increase in prevalence of MIH and highlight the need for increased awareness about this condition among dentists and the general population.

## 1 INTRODUCTION

Molar incisor hypomineralization (MIH) is a developmental defect that has been identified in many countries around the world, with a wide variation in its prevalence (Naysmith & Wm, 2017). MIH has a systemic origin and occurs during the maturation stage of tooth formation (Weerheijm & Jälevik, 2001). It is characterized by an inadequate mineralization of the enamel, affecting one or more of the first permanent molars, and the permanent incisors can be affected as well (Weerheijm & Jälevik, 2001). Teeth with MIH present demarcated opacities that can be chalky white, creamy, yellow, or brown. The identification of demarcated opacities is important for differential diagnosis of other developmental enamel defects, such as dental fluorosis. MIH is a qualitative enamel defect, and its thickness is similar to that of normal enamel (Farah, Drummond, Swain, & Williams, 2010). When the tooth erupts, the enamel is fully formed; this feature differentiates this condition from enamel hypoplasia. However, enamel breakdown may occur after tooth eruption, mainly due to the presence of porous enamel exposed to masticatory forces. Therefore, the appearance of the teeth affected by MIH may deteriorate over time.

The structural defects affecting MIH teeth favor the development of dental caries (Americano, Jacobsen, & Soviero, 2017). Moreover, children with MIH may experience hypersensitivity, and as a result, tooth brushing may be painful. The children with this condition frequently have poor oral hygiene. A literature review of the association of dental caries and MIH concluded that children with MIH were more likely to have dental caries in permanent teeth than were children without MIH (Americano et al., 2017). A study in Finland of the association between dental caries and MIH, considering locality, socioeconomic conditions, age, and MIH as predictors of dental caries, concluded that this enamel defect was the strongest indicator of dental caries in the first permanent molars (Wuollet, Laisi, Alaluusua, & Waltimo‐Sirén, 2018).

There have been efforts to recognize the occurrence and impact of this condition in different countries. In Latin America, several studies indicated the presence of this condition (Jälevik, 2010; Velandia, Álvarez, Mejía, Rodríguez, & De, 2018). The results of a questionnaire offered in 2003 to European pediatric dentists showed that MIH was present in 29 of the 30 European countries included. In this study, the birth cohort was considered a factor explaining the differences in prevalence of MIH (Weerheijm & Mejàre, 2003). Moreover, a study of the perception of academic dental clinicians in a university in the Middle East found that approximately one‐third of the dentists perceived an increased occurrence of this developmental disturbance in permanent teeth (Ghanim, Morgan, Mariño, Manton & Bailey 2011). However, little evidence is available related to the trends over time of MIH prevalence and severity in different population groups. Recognizing trends of this condition could provide information that contributes to the identification of its etiology, an important element in planning dental services and continuing dental education.

The timing of detection and treatment of MIH is critical to prevent children's pain and tooth extraction as much as possible. In the United Kingdom, in dental hospitals, MIH was the second most important cause of extraction of first permanent molars in children, after dental caries (Albadri, Zaitoun, & McDonnell, 2007). Parents' awareness of problems in their children's mouths could encourage them to seek dental treatment and thus a better prognosis of the children's oral health and less negative impact on their quality of life. Prevention of MIH is difficult due to the multiple possible causes related to this condition, such as illness occurring during the first 3 years of childhood life, affecting the mineralization of the teeth; fever, asthma, and pneumonia in particular have been associated with MIH (Silva, Scurrah, Craig, Manton, & Kilpatrick, 2016). This condition has a genetic component, and several genes expressed during enamel formation have been related to MIH (Vieira & Kup, 2016). (Jeremias et al., 2013).

The objectives of the present study were (a) to compare the prevalence of MIH in a group of schoolchildren evaluated in 2008 with a group evaluated in 2017 of the same area of Mexico City, (b) to identify the association of MIH with dental caries in schoolchildren, and (c) to assess the mother's perception of her child's oral health status.

## 2 METHOD

Two cross‐sectional studies were conducted, the first survey in 2008 and the second in 2017. Two public elementary schools were selected for the study; they were located in a low‐income area in southeast Mexico City. In Mexico, there is a National Salt Fluoridation Program, and the children had access to fluoridated salt (Fluor 250 mg/kg). In 2008, a first survey was performed on six‐ to eight‐year‐old children who were born between 2000 and 2002. In the same schools, the second survey took place in 2017. This survey encompassed children born between 2009 and 2011. The same dentist conducted oral examinations in both surveys.

The study protocol was approved by the Ethics Committee of the Autonomous Metropolitan University‐Xochimilco (IRB: CE/12/05/06/1407). The parents were informed of the dental status of their children and the availability of dental clinics where their children could be treated. All the children's parents signed the consent form, and each participating child consented to have an oral examination. The study followed the Declaration of Helsinki norms and those of the local sanitary authorities. A questionnaire was filled out by the mothers and included sociodemographic items and the following question: “Do you consider the oral health condition of your child: good, fair, or poor?”

### 2.1 Index

MIH was evaluated using the European Academy of Paediatric Dentistry Index (Weerheijm, Duggal, Mejàre, et al., 2003). Defects smaller than 2 mm were excluded. A child was classified as having MIH when any of the first permanent molars showed signs of MIH. Based on the European Academy of Paediatric Dentistry index, mild MIH was present when demarcated enamel opacities were observed without posteruptive loss of enamel, and severe MIH when, in addition to demarcated opacities, posteruptive enamel breakdown was observed. One examiner collected the information. For standardization purposes, the examiner used written information and photographs and a 2‐day standardization exercise with children. The group of children examined for calibration purposes were not part of the results of the present study. Dental caries was assessed using the World Health Organization criteria (Organization WH, 1997). The Kappa values for MIH and dental caries were 0.92 and 0.91, respectively. Prior to dental examination, the children brushed their teeth under the supervision of a dentist. A plain mirror and a World Health Organization type of probe were used to eliminate detritus from tooth surfaces, when necessary, to obtain an adequate observation of each tooth surface. Barrier techniques were followed during oral evaluation. The children were in supine position, and artificial white light was used during the examination. The teeth were not dried before examination. The included children had at least one erupted or partially erupted first permanent molar, showing at least half of the clinical crown (Calderara et al., 2005). The exclusion criteria included children with fixed orthodontic appliances that did not allow the evaluation of the tooth surfaces or children who were absent during the days of oral evaluation.

Sample size calculation was performed for a difference in proportions of MIH between surveys of 5%, Proportion_1_ = 0.15 and Proportion_2_ = 0.20, alpha 0.05, and power of 80%. The total sample size was 454, that is, 227 children in each survey. In the first survey, informed consent letters were sent to 265 parents; 259 (97.7%) retuned the letter, accepting their child's participation in the study. In the second survey, the school authorities asked to examine full classes; consequently, 361 children were invited to participate in the study. Consent letters were sent to the parents, and 342 (94.7%) accepted their children' participation. In the first survey, 12 children did not have at least one erupted first permanent molar, and four children were not present during the days of the oral examination. Therefore, the number of children included in the first survey was 232. In the second survey, 19 did not have any permanent teeth with at least half of the clinical crown erupted, and six children were absent on the days when the oral examination was performed. Therefore, the number of children included in the second survey was 317. The sample size by adding both surveys was 549 children.

### 2.2 Statistical analysis

The data was summarized in means and standard deviations, and categorical variables were presented as counts and percentages. The statistical analysis was performed using a logistic regression model and mixed‐effects logistic regression models for the three output variables studied, MIH, dental caries, and maternal perception of a child's dental health, with random intercept, considering that the children were clustered in schools and assuming that the covariance structure was independent. The goodness of fit of the logistic regression models was evaluated using the Hosmer and Lemeshow test. Significance was set at *p* < .05. The STATA V 15 statistical package was used for data analysis.

## 3 RESULTS

A total of 549 children participated in the study, 6–8 years of age, mean age 7.1 (±0.63), and the percentage of girls was 52.2%. These children were selected in two surveys. The first survey was performed in 2008 and included 232 (42.2%) children, and the second survey was carried out in 2017 and included 317 (57.8%) children. The mean age in the first group was 7.0 (± 0.63) and in the second 7.1 (±0.62; *p* = .220). Between the first and the second survey, no significant differences were found by sex, 49.6% and 53.9% girls, respectively (*p* = .311).

### 3.1 Molar incisor hypomineralization

**Figure 1:**
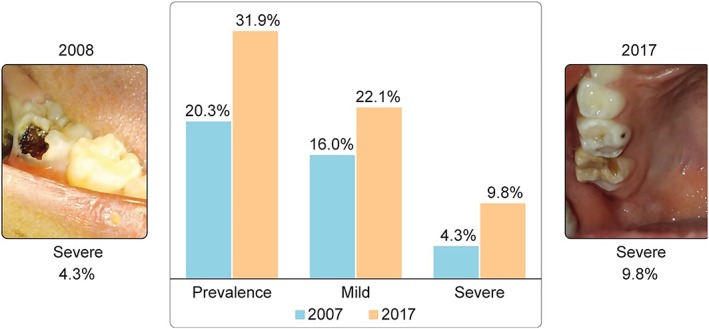
Please center the image in the pagePrevalence and percentage of children in the mild and severe molar incisor hypomineralization categories in 2008 and in 2017

The proportion of children presenting MIH in the first survey was 20.3% and in the second survey 31.9% (*p* = .002). In the first survey, 16.0% of the children were in the mild category and 4.3% in the severe category, and 79.7% of the children did not have MIH. In the second survey, 22.1% were in the mild category, 9.8% in the severe category, and 68.1% of the children did not have MIH (*p* = .005), (Figure 1). In the children presenting MIH in the first survey, the mean number of affected teeth was 2.3 (±1.0), in the second survey, 3.0 (±1.1). Table 1 presents the odds ratio of having MIH, comparing the first and the second surveys. The children in the second survey were more likely to have MIH than the children in the first survey (odds ratio [OR] = 1.85, *p* = .003), adjusted by age (*p* = .755) and sex (*p* = .181).

**Table 1:** Crude and adjusted odds ratios of the mixed‐effects logistic regression model of MIH and year of survey, age, and sex

	Crude OR	(95% CI)	*P*	Adjusted OR^a^	(95% CI)	*P*
Survey						
(Second 2017)	1.84	(1.24 2.74)	.003	1.85	(1.24 2.77)	.003
Age	0.96	(0.71 1.30)	.792	0.95	(0.69 1.30)	.755
Sex (female)	0.80	(0.55 1.16)	.241	0.77	(0.53 1.13)	.181

Abbreviations: CI, confidence interval; OR, odds ratio.

a OR adjusted for age and sex. Reference category: sex = male, survey = first survey (2008). Random intercept School (variable) 0.01, 95% CI (0.001 5.94)

Considering both surveys, maternal perception of the children's oral health showed that 27.0% of the mothers perceived their child's oral condition to be good, 58.3% fair, and 14.7% poor. Table 2 presents the distribution of maternal perception by MIH status in each survey. Maternal perception was similar in both surveys. Approximately 10% of the mothers of children with MIH perceived that the oral health of the child was good, and around a third of the mothers had this perception in children without MIH. Both the first (*p* = .010) and second surveys (*p* < .0010) showed an association between MIH and maternal perception of a child's oral health status.

**Table 2:** Distribution of sociodemographic characteristics by the presence of MIH in the first survey and the second survey

First survey (year 2008)	MIH *n* = 47	No MIH *n* = 185	*P*
Age mean (SD)	7.9 (0.54)	8.0 (0.66)	.088
Sex			
female *n* (%)	19 (16.5)	96 (51.9)	.160
male *n* (%)	28 (23.9)	89 (48.1)	
Maternal perception of child's oral health			
good *n* (%)	5 (10.6)	60 (32.4)	.010
fair *n* (%)	36 (76.6)	103 (55.7)	
poor *n* (%)	6 (12.8)	22 (11.9)	

Second survey (year 2017)	MIH *n* = 101	No MIH *n* = 216	
Age mean (SD)	8.1 (0.65)	8.1 (0.60)	.433
Sex			
female *n* (%)	52 (51.5)	119 (55.1)	.548
male *n* (%)	49 (48.5)	97 (44.9)	
Maternal perception of child's oral health			
good *n* (%)	10 (9.9)	73 (33.8)	˂.001
fair *n* (%)	70 (69.3)	111 (51.4)	
poor *n* (%)	21 (20.8)	32 (14.8)	

Abbreviations: MIH, molar incisor hypomineralization; SD, standard deviation.

### 3.2 Dental caries

In the first survey, in primary teeth, the mean decayed, missing, or filled teeth (dmft) was 4.84 (±5.11), and in the second survey, the dmft was 4.30 (±3.49), (*p* = .1417). In the first survey, the most important component was decayed teeth, *d* = 3.48 (± 4.56) conforming to 72.0% of the index, followed by filled teeth 24.8%; *f* = 1.20 (± 1.99), and the least important component was missing teeth 3.2%, m = 0.16 (± 0.48). A similar composition was observed in the second survey: 73.5%, d = 3.16 (± 3.02), followed by filled teeth 23.0%; f = 0.99 (± 1.74), and the lowest component derived from missing teeth 3.5%; *m* = 0.15 (± 0.53). The difference in the components between years of survey was not statistically significant (*p* > .05).

The permanent dentition, the mean DMFT, was 0.46 (±1.4) and 0.39 (±0.98) in the first and second surveys (*p* = .530), respectively. Considering only the first permanent molars, the DMFT = 0.44 (±1.4) in the second survey and DMFT = 0.38 (±0.92). As observed in the primary dentition, the most important component of the DMFT index was decayed teeth followed by filled teeth; in addition, there were no missing permanent teeth in either of the two surveys. In the first survey, 60%; *D* = 0.26 (±1.08) corresponded to decayed teeth; in the second, 86.8%, *D* = 0.33 (±0.77), (*p* = .016), corresponded to decayed teeth. Filled teeth corresponded to 40%, *F* = 0.18 (±0.85), and 13.2%, *F* = 0.05 (±0.44), in the first and second surveys, respectively (*p* = .004).

Table 3 presents the caries index by MIH status in the first and second surveys. In the first survey, among the children with MIH, 29.8% had dental caries in their permanent teeth; among the children without MIH, this percentage was 17.8% (*p* = .069). In the second survey, among the children with MIH, 36.6% had dental caries in their permanent teeth; among the children without MIH, this percentage was 13.9% (*p* < .001). No significant association was found between the presence of MIH and dental caries in the primary teeth. Considering the mean of the dental caries index, in the first survey, the mean dmft was 5.02 (±4.96) and 4.79 (±5.16) in the groups with MIH and without MIH (*p* = .787), respectively. In the second survey, the mean dmft was 4.75 (±3.53) and 4.08 (±3.46) in the groups with MIH and without MIH (*p* = .114), respectively. In permanent dentition, in the first survey, the mean DMFT was 0.63 (±1.72) in the group with MIH, and DMFT was 0.39 (±1.25) in the group without MIH (*p* = .263). In the second survey, a significant difference was detected between the mean dental caries and MIH status. The DMFT was 0.61 (±0.99) in the group with MIH, and in the group without MIH, the DMFT was 0.26 (±0.86), (*p* = .002). Table 4 presents the OR of the mixed‐effects logistic regression model for dental caries and MIH, controlled by age and sex. In both surveys, MIH was associated with the presence of dental caries in the first permanent molars; the ORs were 2.24 (*p* = .036) and 4.18 (*p* ˂ .001) in the first and second surveys, respectively. In the second survey, the clustering effect of the school was significant (*p* = .040).

**Table 3:** Number and percentage of children with dental caries in primary and permanent teeth by the presence of molar incisor hypomineralization in the 2008 and the 2017 surveys

First survey (2008)	DMFT = 0 *n* (%)	DMFT ≥ 1 *n* (%)	*P*	DMFT = 0 *n* (%)	DMFT ≥ 1 *n* (%)	*P*
MIH yes *n* = 47	13 (20.7)	34 (20.1)	.931	33 (17.8)	14 70.2	.069
MIH no *n* = 185	50 (79.3)	135 (79.9)		152 (82.2)	33 (29.8)	
**Second survey (2017)**						
MIH yes *n* = 101	12 (23.5)	89 (33.5)	.163	64 (25.6)	37 (44.8)	˂.001
MIH no *n* = 216	39 (76.5)	177 (66.5)		186 (74.4)	30 (55.2)	

Abbreviations: DMFT, decayed, missing, or filled teeth; MIH, molar incisor hypomineralization.

**Table 4:** Odds ratios of the mixed‐effects logistic regression model of dental caries in first permanent molars by year of survey

First survey (2008)	Odds ratio	95% (CI)	*P*
MIH	2.24	(1.06 4.78)	.036
Age	1.87	(1.13 3.10)	.015
Sex (female)	0.96	(0.50 1.86)	.911
**Second survey (2017)**			
MIH	4.18	(2.21 7.91)	˂.001
Age	1.04	(1.13 3.10)	.327
Sex (female)	1.88	(0.99 3.59)	.054

*Note.* First survey, random intercept: school (variable) 0.0001. Likelihood‐ratio test versus logistic model: *χ* ^2^ *p* ˂ 0.99. Second survey, random intercept: school (constant) 2.89, 95% CI (0.51 16.45).

Abbreviations: CI, confidence interval; MIH, molar incisor hypomineralization.

The results of the mixed‐effects model for maternal perception of a child's oral health and dental condition are presented in Table 5. Maternal perception of a child's poor oral health was associated with higher dental caries (dmft + DMFT) indices (OR = 1.20, *p* ˂ .001); similarly, poor maternal perception of children's oral health status was also associated with MIH (OR = 4.47, *p* ˂ .001), adjusted by age, sex, and year of the survey.

**Table 5:** Crude and adjusted odds ratios of the mixed‐effects logistic regression models of maternal perception of a child's oral health and sociodemographic, dental caries, and molar incisor hypomineralization

	Crude OR	(95% CI)	*P*	Adjusted OR^a^	(95% CI)	*P*
Age	0.88	(0.65 1.19)	.261	0.89	(0.64 1.25)	.516
Sex (female)	1.16	(0.79 1.69)	.443	1.24	(0.82 1.86)	.309
Survey Second (2017)	1.09	(0.74 1.60)	.661	1.08	(0.70 1.65)	.740
Dental Caries (DMFT + DMFT)	1.20	(1.13 1.27)	˂.001	1.20	(1.13 1.28)	˂.001
MIH (yes)	4.38	(2.47 7.78)	˂.001	4.47	(2.45 8.14)	˂.001

Abbreviations: CI, confidence interval; DMFT, decayed, missing, or filled permanent teeth; MIH, molar incisor hypomineralization. OR, odds ratio.

a OR adjusted for age, sex, maternal education and maternal oral health perception. Reference category: sex = male, survey = first survey (2008). Base outcome good and fair maternal perception of her child oral health. Random intercept: school (constant) 0.41, 95% CI (0.03 5.13).

## 4 DISCUSSION

The comparison of the two cross‐sectional surveys analyzed in this study showed a higher MIH prevalence in 2017 than in 2008. In addition, the percentage of MIH cases classified as severe was also higher in the second survey than in the first survey. Since the early 2000s, attention has been drawn to the possibility of an increased incidence of this condition in Europe and in other regions (Jälevik, 2010; Ghanim et al., 2011; Weerheijm & Mejàre, 2003). The reason for the increment in MIH found in the Mexican children is unclear. It may be related to the increase in concentration of pollutants in our modern environments.(Crombie, Manton, & Kilpatrick, 2009) Ameloblasts are very sensitive to environmental factors during the maturation phase (Suga, 1989). In an archeological case series study, MIH was found to be practically nonexistent, and it was suggested that MIH may be linked to contemporary living conditions (Kühnisch et al., 2016).

There are questions about MIH etiology; for example, it may be from systemic changes produced by childhood illness, the drugs administered for the treatment for this illness, or both (Silva et al., 2016). Some studies associated MIH with the use of antibiotics at an early age (Hysi et al., 2016; Laisi et al., 2009). In Mexico in 2010, a prohibition of over‐the‐counter sales of antibiotics was legislated; since then, a prescription has been required, (Dreser, Vázquez‐Vélez, Treviño, & Wirtz, 2012) and the sales of antibiotics dropped after this regulatory change (Santa‐Ana‐Tellez, Mantel‐Teeuwisse, Dreser, Leufkens, & Wirtz, 2013). Most of the children examined in the second survey lived their first years of life after this legislation was enforced, unlike the children in the first survey. However, no decrease in MIH was found. Nevertheless, the private sector, to facilitate the prescription of these drugs, installed physicians' offices right next to the pharmacies, and patients could go to these offices and obtain a prescription. It is possible that mothers of the children in the present study took them to these medical offices when the child was sick and antibiotics were prescribed. These offices have low consultation fees (less than $2), and some are free of charge. In addition, antibiotics could be present in small quantities in food. For example, in the main brands of powdered milk sold in Mexico City, the presence of residues of antibiotics was found (Kneebone & W C, 2010). Longitudinal studies are required to ascertain the causes of MIH that could explain its possible increment.

The index used in this study was the European Academy of Paediatric Dentistry MIH Index, which has good sensitivity and specificity (Ghanim, Mariño, & Manton, 2019). The same indices were used in the first and second surveys; the same schools were selected, and one examiner performed the oral evaluations in both surveys. To reduce information bias, the assessment of dental caries and MIH were carried out separately and were recorded in different charts. However, considering that the same examiner preformed both assessments, information bias cannot be ruled out. It is also possible that the presence of restorations could mask the occurrence of MIH; however, in the present study, few children had restorations in their permanent teeth.

The study group encompassed low‐income families; this limits the extrapolation of the findings to higher‐income groups. However, a large number of Mexico City residents have low income. The association between MIH and socioeconomic conditions is unclear. A study in northern England, in fluoridated and nonfluoridated communities, found that children from high‐income families were more likely to have MIH than children in quintiles with greater deprivation; no association was observed from the exposure to fluoridated water (Balmer, Toumba, Godson, & Duggal, 2012). In contrast, a study in Brazil found that children from schools in rural areas were more likely to have MIH than were their counterparts in urban areas (da Costa‐Silva et al., 2010). More studies are required to investigate the association of socioeconomic conditions and occurrence of MIH.

There is little information on the prevalence of MIH in North America; in Mexico, a prevalence of 15.6% was found. In this previous study, the year in which it was carried out was not identified. The prevalence was lower than the one observed in the present study (Gurrusquieta et al., 2017). Several factors are likely to be associated with the etiology of MIH that could contribute to differences in the prevalence of this condition in different groups in the same country. However, variations in methodological aspects could limit the comparisons of prevalence of MIH among studies. The frequency of MIH was greater in molars than in incisors. It is known that the thicker the enamel, the longer the period was that the tooth was exposed to insults; this may explain the higher prevalence of MIH in molars than in anterior teeth.

In this study, an association was found between dental caries in permanent teeth and the presence of MIH, in both surveys. A systematic literature review recognized a significant association between dental caries in permanent teeth and the presence of MIH. Children with MIH were more likely to have dental caries than children without MIH. The ORs found in the present study were in the range of those identified in this review (Americano et al., 2017). The enamel of MIH teeth has a lower mineral content and has more protein than sound enamel, and biofilm‐retentive areas were observed in the parts of the tooth with broken enamel. These factors contribute to the higher risk of dental caries observed in MIH teeth (Americano et al., 2017).

In the present study, greater severity of MIH was observed in the second survey. Higher MIH severity is associated with higher prevalence of dental caries (Gurrusquieta et al., 2017). Severely affected MIH teeth are difficult to treat as the child may experience hypersensitivity, and it may be difficult to obtain analgesia (da Cunha Coelho et al., 2019). In addition, the restoration of the tooth may be technically challenging due to the likelihood of enamel breakdown. The high porosity of the enamel, in turn, affects the longevity of the restorations. Moreover, severely affected teeth frequently require retreatment and may need to be extracted (Oliver et al., 2014).

No evidence of a decrease in dental caries was observed between the surveys, which were performed with a 9‐year interval between them. It is possible that the increased presence of MIH limited the decline in dental caries, which was the aim of the National Salt Fluoridation Program. In both surveys, the main component of the index was decayed teeth. With respect to permanent dentition, fewer teeth were filled in the second survey than in the first survey. The area studied was hit by the 2017 Mexico City earthquake. The schools included were in low‐income areas, and many families suffered severe destruction of their homes; several streets were closed for months. We speculate that this could have affected the pursuit of dental treatment for the children.

MIH was associated with a more negative maternal perception of a child's oral health. Although the recognition of poor dental health in children is important, it is unclear how this perception is processed in the parents. Further research is required to investigate the dynamics of their coping mechanisms (Nelson et al., 2015). The parents' recognition of their children's oral health problems is a basic step toward the search for treatment. However, barriers such as large distances to access dental care facilities and limited public dental services have made it difficult for low‐income families to take care of children with MIH. Parents who do not recognize their children's health problems may be less likely to facilitate opportune visits to the dentist. Children with severe MIH are known to visit a dentist up to 10 times more than the children without this condition (Jälevik, 2002).

In a study of the burden of MIH in the world, it was estimated that the number of MIH cases was about 900 million in 2015 (Schneider & Silva, 2018). It is necessary to raise the awareness of the public, government authorities, and dental health professionals, particularly pediatric dentists, to give the due attention this condition requires.

## 5 CONCLUSION

Higher prevalence and severity of MIH was found in 2017 than in 2008. It is possible that the presence of MIH limited the decline in dental caries. Dental professionals should be aware of the presence of MIH and its possible increase in the young population, in order to detect it as soon as possible and be prepared to provide suitable treatment to improve the quality of life of the patients suffering from MIH.
